# Spotting the Diagnosis: A Case Report of Paraneoplastic Retinopathy Masquerading As White Dot Syndrome

**DOI:** 10.7759/cureus.115030

**Published:** 2026-08-23

**Authors:** Aakanksha Gundu, Henry Holt, Ann Ostrovsky

**Affiliations:** 1 Division of Ophthalmology, East Carolina University Brody School of Medicine, Greenville, USA; 2 Division of Ophthalmology, East Carolina University Health Medical Center, Greenville, USA

**Keywords:** multidisciplinary follow-ups, multiple sclerosis, ocular oncology, paraneoplastic retinopathy, relentless placoid chorioretinitis, white dot syndromes

## Abstract

This study aimed to report a rare case of presumed paraneoplastic retinopathy in a patient with multiple sclerosis who initially presented with clinical findings suggestive of white dot syndrome. This report describes a complex neuro-ophthalmic presentation where initial diagnostic considerations were reframed to reach the final diagnosis. A 46-year-old female with a past medical history of multiple sclerosis presented with progressively worsening vision in the left eye, a central blind spot, and visual disturbances. Initially, her symptoms were attributed to multiple sclerosis-associated optic neuritis or a white dot syndrome. However, the patient’s atypical clinical course and lack of response to treatment broadened the differential diagnosis to include masquerade syndromes. The inconclusive results of a vitrectomy, worsening vision, new involvement of both eyes, and lack of response to traditional treatment ultimately led to a presumed diagnosis of paraneoplastic retinopathy. This case study informs ophthalmologists about ocular conditions with similar presentations and emphasizes the importance of maintaining a broad differential diagnosis, conducting diligent investigation, and engaging in multidisciplinary collaboration when faced with complex neuro-ophthalmic presentations.

## Introduction

Paraneoplastic retinopathy is a rare paraneoplastic syndrome that affects the visual and nervous systems and occurs in the setting of malignancy. Specifically, it represents a nonmetastatic distant consequence of an underlying malignancy. It causes visual deterioration and worsening visual acuity due to molecular mimicry of retinal cells by antigens on tumor cells, leading to immune-mediated cross-reactivity that damages normal tissues [1]. Despite ocular immune privilege mechanisms, such as the blood-retinal barrier and an immunosuppressive microenvironment, autoimmune conditions may still produce ocular manifestations [2]. While paraneoplastic retinopathy can indicate disease progression in 20% of cases, it is more commonly the initial presentation in 70% of cases, often presenting months before detection of the underlying malignancy [1,3]. Paraneoplastic retinopathy requires prompt diagnosis and management to prevent further retinal damage and preserve visual function [1,3].

This case highlights the challenges of diagnosing ocular phenotypes consistent with paraneoplastic retinopathy. It underscores the importance of ongoing investigation and a comprehensive differential diagnosis, especially due to clinical overlap with systemic autoimmune conditions, white dot syndromes, posterior uveitis, and other antibody-mediated diseases.

This project was previously presented in part as a poster at the North Carolina Society of Eye Physicians and Surgeons Annual Meeting on September 14, 2024.

## Case presentation

A 46-year-old female with a past medical history of multiple sclerosis (MS) presented with progressively worsening vision and a central blind spot in her left eye. Her symptoms had begun seven months prior and included left-sided blurry vision, radiating pain from her occipital region, floaters, flashes localized to the left eye, and decreased vibrancy of colors. Her past ocular history was significant for left optic neuritis and a working diagnosis of multiple evanescent white dot syndrome, which a previous ophthalmologist had attributed to numerous unilateral retinal white dots in the left eye. One month before presentation, her MS treatment was transitioned from diroximel fumarate to ofatumumab due to escalating systemic complaints of left-sided weakness and vision loss, along with progressive right-sided lesions on MRI of the brain.

**Figure 1:**
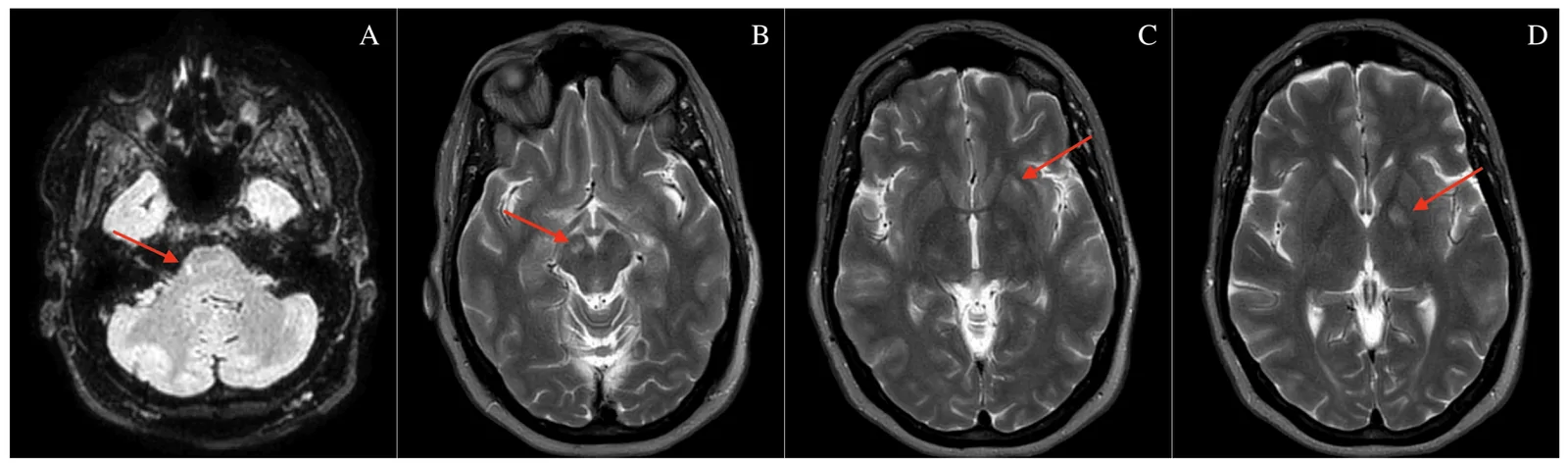
MRI of the brain and orbits without contrast at presentation. Imaging showed progressive scattered hyperintense lesions, measuring up to 1.0 cm, in the right hemi-pons (A), right cerebral peduncle (B), left putamen (C), left globus pallidus (D), and supratentorial white matter. Arrows indicate the lesions detected on MRI.

**Figure 2:**
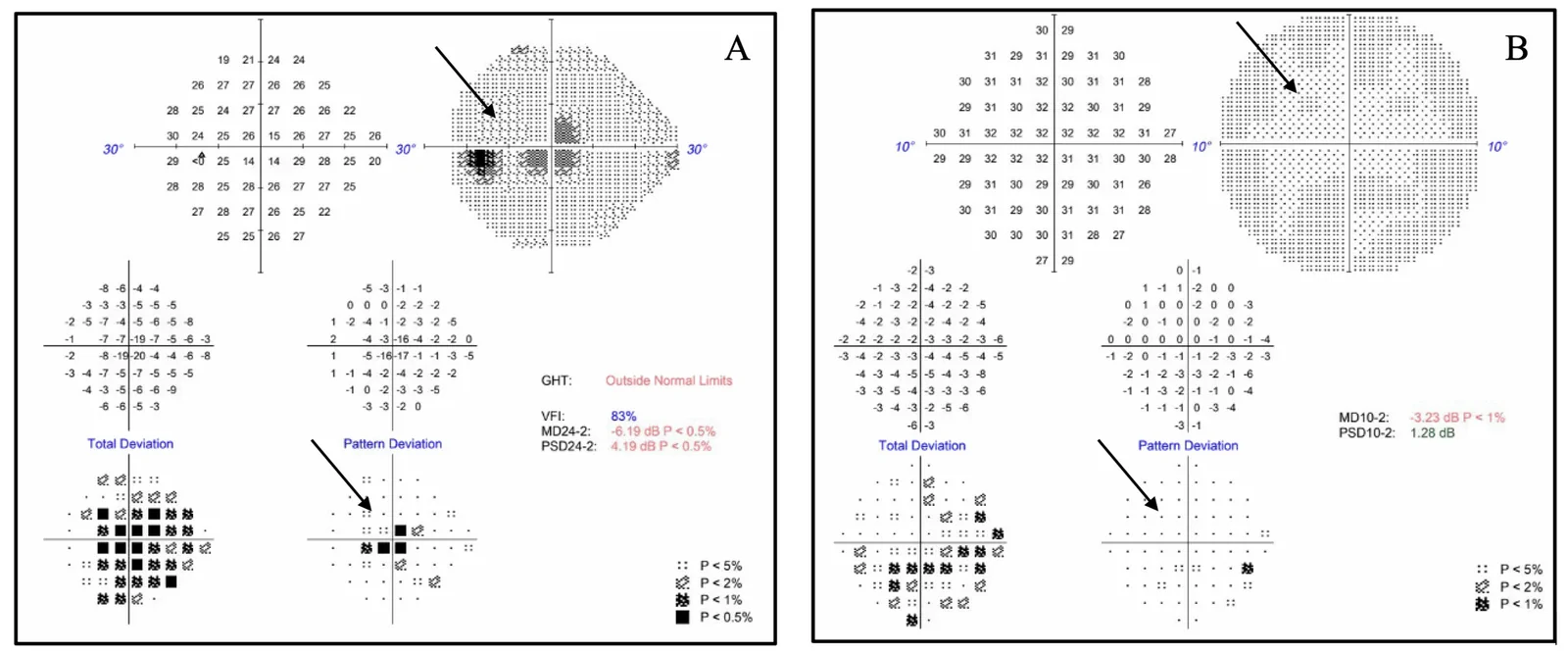
Humphrey visual field. At presentation, a central scotoma was present in the left eye (A). Five months later, visual field testing showed moderate improvements in the left eye (B). Arrows indicate the central scotoma and visual field defects at presentation (A) and resolution of the visual field defects (B).

**Figure 3:**
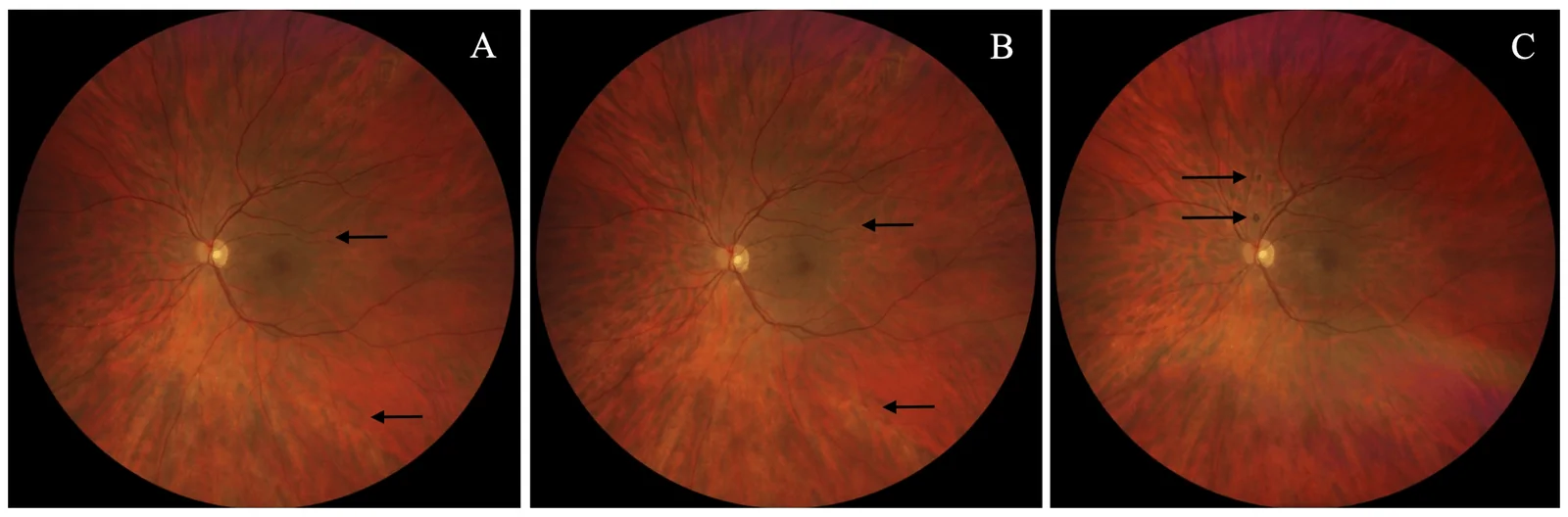
Fundoscopic examination. Left eye at initial presentation (A) and two months later (B), showing subtle central and peripheral small white dots. (C) Eighteen months after presentation, fundoscopy revealed additional pigmentary changes of the supranasal macula in the left eye. Arrows indicate white dots (A, B) and pigmentary changes (C).

**Figure 4:**
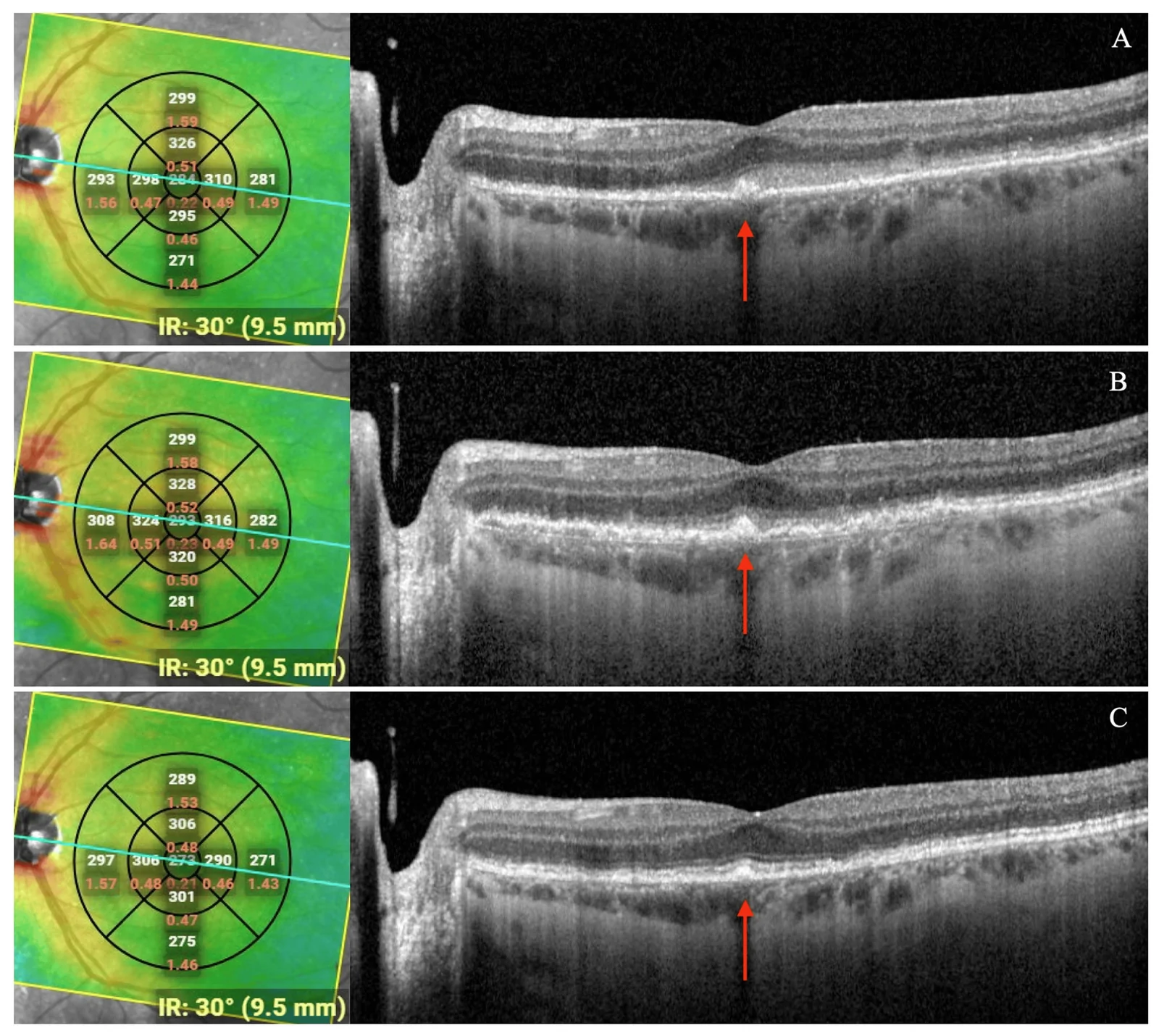
Spectralis optical coherence tomography (OCT) of the left eye. Initial presentation showed an intact external limiting membrane, disrupted ellipsoid zone, granular and thickened hyperreflective retinal pigment epithelium (RPE), with nodular elevations in the outer central macula (A). Two months after initial presentation, worsening vision with worsening outer retinal defects were observed (B). After mycophenolate mofetil treatment and five months after initial presentation, imaging showed improved outer retinal defects (C). Arrows indicate nodular elevations and changes in the RPE throughout the patient's clinical course.

Brain and orbital MRI at presentation demonstrated a stable T2 hyperintensity of the left optic nerve and a normal, symmetric appearance of the orbits, optic nerves, optic chiasm, and post-chiasmatic optic tracts. Progressive scattered T2/fluid-attenuated inversion recovery (FLAIR) hyperintense lesions, measuring up to 1.0 cm, were observed in the right hemi-pons, right cerebral peduncle, left putamen, left globus pallidus, and supratentorial white matter (Figure 1). An ophthalmic exam revealed a best-corrected visual acuity of 20/20 OD and 20/70 OS. The anterior segment examination was unremarkable bilaterally. There was 1+ posterior vitreous cell without snowballs or snowbanking in the left eye. Visual field testing revealed a central scotoma (Figure 2A), a mild afferent pupillary defect, and significant color vision loss (1/10 Ishihara) in the left eye. A fundoscopic examination of the left eye showed small white dots in the peripheral retina and posterior pole with involvement of the macula (Figure 3A). The retinal nerve fiber layer was full in both eyes, but the ganglion cell complex was thinned more prominently in the left eye (OS) compared to the right eye (OD). Spectralis optical coherence tomography (OCT) imaging revealed an intact external limiting membrane, a disrupted ellipsoid zone, and a granular, thickened, hyperreflective retinal pigment epithelium (RPE) with nodular elevations in the outer central macula (Figure 4A) of the left eye. The right eye’s physical exam findings and imaging were unremarkable. The patient was referred to a retinal specialist, who attributed the visual symptoms and findings to MS and prior left optic neuritis, recommending observation.

**Figure 5:**
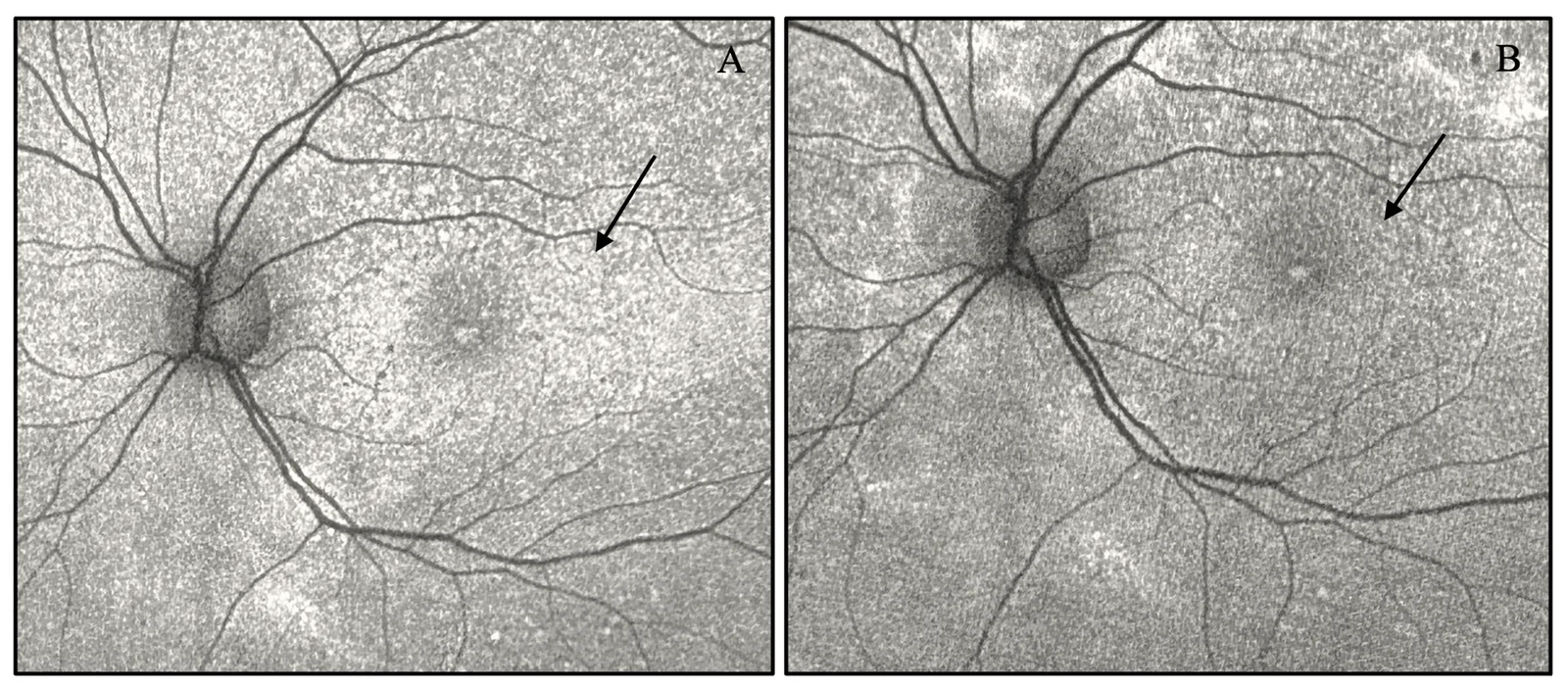
Fundus autofluorescence of the left eye. Initial presentation with a large central macula placoid lesion (A) vs. improved placoid changes after starting mycophenolate mofetil (B) are depicted. Arrows indicate the placoid lesion in the macular region before and after mycophenolate mofetil treatment.

The patient returned two months later with complaints of worsening vision, and her best-corrected visual acuity had decreased to 20/100 OS. A fundoscopic examination of the left eye showed stable white dots in the posterior pole and peripheral retina (Figure 3B). OCT imaging of the left eye revealed more prominent outer retinal defects (Figure 4B). Autofluorescence imaging showed a large central macular placoid lesion in the left eye (Figure 5A), and intravenous fluorescein angiography revealed a few pinpoint areas of mild leakage in the left eye. Repeat MRI of the brain and orbits revealed stable findings, with no increased enhancement of the left optic nerve. The right eye’s exam and imaging remained unchanged. Laboratory evaluation for syphilis, tuberculosis, and Lyme disease antibodies was repeatedly negative. A lumbar puncture was performed as part of her neurological evaluation and demonstrated the presence of oligoclonal bands. The patient was started on high-dose prednisone (1 mg/kg) and eventually transitioned to mycophenolate mofetil. Her visual acuity improved to 20/50 OS within a few weeks. However, mycophenolate mofetil was soon discontinued due to blood dyscrasia, and a dexamethasone implant was placed. Best-corrected visual acuity continued to improve to 20/30 OS. Fundus autofluorescence of the left eye showed a significant improvement in the placoid lesion (Figure 5B), and visual field testing showed improvement of the central scotoma (Figure 2B). At this time, a diagnosis of relentless placoid chorioretinitis (RPC) was made.

However, two months after the dexamethasone implant, the patient returned with decreased visual acuity and was referred to an ocular oncologist. A fundus examination of the left eye showed superior multifocal yellow-white deep lesions with indistinct borders. OCT imaging revealed sub-RPE hyperreflective lesions with mild overlying subretinal fluid and an attenuated external limiting membrane layer in the left eye. Autofluorescence imaging showed speckled hyperautofluorescence superior to the nerve and subtle hypoautofluorescence mottling in the macula and periphery of the left eye. Indocyanine green angiography revealed multiple scattered hypocyanescent spots in the left eye. Fluorescein angiography showed late peripheral perivascular leak and speckled staining in the mid-periphery. Due to the lack of response to conventional therapy, the ocular oncologist concluded that the findings were inconsistent with MS-associated uveitis or typical white dot syndromes. The differential was expanded to include myelin oligodendrocyte glycoprotein antibody-associated disease, neuromyelitis optica spectrum disorder, and masquerade syndromes, specifically intraocular lymphoma and paraneoplastic retinopathy.

The patient’s vision continued to worsen and now involved both eyes, with a best-corrected visual acuity of 20/126 OD and 20/40 OS. A retinal specialist was consulted, and a dexamethasone implant was placed in the right eye. Visual acuity improved from 20/126 to 20/40 in the right eye. Disease progression included bilateral posterior uveitis, bilateral vitreous detachments, bilateral disseminated chorioretinal inflammation, and a vitreomacular adhesion in the right eye. Fundus photography revealed new subretinal white lesions of the temporal macula in the right eye and pigmentary changes of the superonasal macula in the left eye (Figure 3C). OCT of the retina showed reduced integrity of outer bands, sub-RPE elevation, and subretinal hyperreflective material in the right eye, and stable sub-RPE elevation in the left eye (Figure 4C). Autofluorescence showed a bilateral “leopard spot” appearance. Slit-lamp examination of the right eye revealed a posterior vitreous detachment with 1+ vitreous cells; the left eye demonstrated 2+ vitreous cells associated with multifocal yellow lesions and sub-RPE amelanotic deposits. Repeat brain MRI showed unchanged tiny foci of T2/FLAIR hyperintensity in the supratentorial and infratentorial white matter, presumed to be related to the patient’s history of MS, with no evidence of active demyelinating disease or new white matter lesions. A pars plana vitrectomy in the right eye was inconclusive for ocular lymphoma. The patient’s clinical course, characterized by corticosteroid interference and the common presentation of ocular symptoms before primary malignancy detection, led to a diagnosis of presumed paraneoplastic retinopathy. Following the initiation of intravitreal rituximab, visual acuity in the right eye improved to 20/50 with pinhole acuity of 20/30. To identify a potential occult primary tumor, the patient remains under close surveillance by her ophthalmology and neurology teams to undergo an extensive malignancy workup, including computed tomography, positron emission tomography scans, and electroretinography.

## Discussion

The intricate relationship between systemic neurological disorders and ocular manifestations often poses considerable diagnostic and therapeutic challenges in clinical practice. Differentiating between primary ocular disease and manifestations of systemic conditions requires a comprehensive diagnostic approach. This case is unusual due to the atypical and progressive nature of the patient’s ocular symptoms despite a known MS diagnosis, which led to an initial misleading diagnosis of RPC.

RPC, or ampiginous choroiditis, is a rare, bilateral disease of the RPE and choroid. Due to disease overlap and similar presentation as serpiginous choroiditis (SC) and acute posterior multifocal placoid pigment epitheliopathy (APMPPE), RPC is often misdiagnosed [4]. These disorders are classified as white dot syndromes, or inflammatory choroidopathies causing white lesions at the deeper levels of the retina and choroid. Although prior research suggests potential viral, bacterial, or autoimmune etiologies of white dot syndromes, a definitive cause is still unknown [4]. Patients initially present with blurred vision, visual field loss, photopsia, floaters, and scotomas. Overall, white dot syndromes are differentiated based on the size and shape of the chorioretinal lesions. Unlike APMPPE and SC, RPC is characterized by a disease course exceeding six months and lesions distributed in the mid and far periphery [5]. Treatment for white dot syndromes consists of local or systemic corticosteroids with a transition to steroid-sparing systemic immunotherapy [4]. Immunotherapy must be carefully monitored as white dot syndromes may be associated with autoimmune diseases.

Vitreoretinal lymphomas, a known masquerader of white dot syndromes, mimic chronic posterior uveitis, respond to initial steroid therapy, and are diagnosed using cytology to identify lymphoma cells in the eye [6]. Vitreoretinal lymphomas present with yellowish retinal or subretinal infiltration, optic disc swelling, and occasionally retinal vasculitis [7]. They are often difficult to differentiate from intraocular inflammatory diseases due to symptomatic overlap [7]. Furthermore, a negative vitrectomy with limited cells due to previous corticosteroid treatment is a commonly encountered obstacle in diagnosis, as seen in this case. Spontaneous resolution of subretinal lesions and absent vitreous inflammation may limit the vitreous tap [6]. Masquerading syndromes often delay the definitive diagnosis; therefore, it is important to thoroughly investigate the clinical course and remain curious, a trait that allowed the ophthalmologists in this case to consider paraneoplastic retinopathy.

Paraneoplastic retinopathy is a clinical diagnosis traditionally supported by unexplained progressive visual loss, the presence of serum anti-retinal antibodies, and an underlying systemic malignancy [8]. The diagnostic workup often relies on electroretinography, which classically demonstrates early and marked attenuation of photoreceptor responses [9], alongside the detection of circulating anti-retinal autoantibodies [8]. This patient’s clinical presentation fits a presumed paraneoplastic etiology due to the relentless, bilateral progression of visual decline, the presence of atypical chorioretinal lesions, and the ultimate lack of response to potent immunosuppression. Furthermore, paraneoplastic retinopathy has been described as a nonmetastatic remote effect of carcinoma that frequently presents in postmenopausal women [10]. This demographic precedent aligns well with the patient's hormonal and clinical profile, further supporting the presumed diagnosis despite the initial atypical presentation. The patient’s initial presentation with a central scotoma also closely aligns with established clinical characterizations of the disease. In their extensive clinical profiling of paraneoplastic retinopathy, Makiyama et al. demonstrated that scotomas and visual field defects are frequently observed visual disturbances at presentation, as seen in this patient, further corroborating the presumed diagnosis despite the initial masking by the white dot syndrome phenotype [9]. This case deviates from a classic presentation as the primary malignancy has not yet been identified on imaging, and the initial unilateral presentation mimicking a white dot syndrome is highly atypical. However, because ocular symptoms can precede the detection of a primary tumor by months or even years [1,3,8], the diagnosis remains presumed paraneoplastic retinopathy pending the results of her ongoing systemic surveillance.

## Conclusions

Despite a prior diagnosis of MS and initial considerations aligning with MS-related ocular inflammation and rare conditions, including multiple evanescent white dot syndrome and RPC, the patient’s persistent and atypical progression necessitated a comprehensive and detailed re-evaluation. The evolving clinical picture, characterized by a lack of clear response to traditional anti-inflammatory treatments and subsequent involvement of both eyes, ultimately led to the presumed diagnosis of an ocular phenotype suspicious for paraneoplastic retinopathy. This case highlights the importance of maintaining a broad differential diagnosis, ongoing persistent investigation, and multidisciplinary collaboration when faced with complex neuro-ophthalmic presentations.

## References

[1] Duncan C, Zamir E, Teh J, Joon DL, Liodakis P (2019). A blind spot in urology: prostate cancer associated retinopathy. Urol Case Rep.

[2] Zhou R, Caspi RR (2010). Ocular immune privilege. F1000 Biol Rep.

[3] Rahimy E, Sarraf D (2013). Paraneoplastic and non-paraneoplastic retinopathy and optic neuropathy: evaluation and management. Surv Ophthalmol.

[4] Yeh S, Lew JC, Wong WT, Nussenblatt RB (2009). Relentless placoid chorioretinitis associated with central nervous system lesions treated with mycophenolate mofetil. Arch Ophthalmol.

[5] Mirza RG, Jampol LM (2012). Relentless placoid chorioretinitis. Int Ophthalmol Clin.

[6] Sheth N, De Alba M, Challa N, Massengill MT, Mieler WF (2025). Diagnosis of secondary vitreoretinal lymphoma with neurosurgical stereotactic biopsy: a multimodal diagnostic and imaging approach. J Vitreoretin Dis.

[7] Ishida T, Takase H, Arai A, Ohno-Matsui K (2020). Multimodal imaging of secondary vitreoretinal lymphoma with optic neuritis and retinal vasculitis. Am J Ophthalmol Case Rep.

[8] Adamus G (2009). Autoantibody targets and their cancer relationship in the pathogenicity of paraneoplastic retinopathy. Autoimmun Rev.

[9] Makiyama Y, Kikuchi T, Otani A (2013). Clinical and immunological characterization of paraneoplastic retinopathy. Invest Ophthalmol Vis Sci.

[10] Adamus G (2015). Latest updates on antiretinal autoantibodies associated with vision loss and breast cancer. Invest Ophthalmol Vis Sci.

